# Book Reviews

**Published:** 1904-12

**Authors:** 


### BOOK REVIEWS.

The Surgical Treatment of Bright's Disease.—By George M. Edebohis, A. M., M. D., LL. D., professor of the diseases of women in the New York Post Graduate Medical School and Hospital. This volume is intended to shed light upon the new, operative treatment of so fatal a malady as chronic nephritis, and its results.

For a complete systematic presentation of the subject the author thinks the time not ripe.

Seventy-two cases of operation are detailed, and of these it is claimed thirteen patients received no benefit, while fifty-nine experienced amelioration varying from slight and temporary improvement to complete cure.

These results the author believes establish surgery as at present the main, if not the only hope, of sufferers from a hitherto incurable malady. (F. F. Lisiecki, 9 Murray St., N. Y. Octavo. 331 pages, price $2.00.)

The Internal Secretions and the Principles of Medicine.—By Charles E. de M. Sajous, M. D., Fellow of the College of Physicians of Philadelphia, etc., etc. Volume First, with forty-two illustrations. F. A. Davis Co., Philadelphia, publishers, 1903. 800 pages.

To say that this book is the result of most thorough research and careful compilation, and that it is exceptionally well gotten out mechanically, containing some of the best illustrations recently appearing in a medical work, is giving but a meagre idea of its value and its appearance.

Dr. Sajous' work as a writer is well known and this volume is fully up to his usual standard.

The twelve chapters in this first volume cover, Physiology of the Adrenals, viewed from the standpoint of Clinical Pathology—The Internal Secretions of the Adrenals in its Relations to the Respiratory Processes and the Composition of the Blood—In its Relations to the General Oxidation Processes—The Internal Secretions of the Thyroid and Thymus Glands in their Relations to the Adrenals—The Anterior Pituitary Body—The Thyroid Gland, and the Adrenals as Parts of an Autonomous System—The Adrenal System and Vasomotor Functions—The Adrenal System, the General Motor System, and the Pneumogastric Nerve—The Internal Secretions of the Pancreas and Spleen—The Adrenal and Vagal Systems in their Relations to Cardiac and Pulmonary Functions—The Posterior Pituitary as the Functional Center of the Nervous System, and as the Anterior Pituitary's Co-center in sustaining the Vital Processes—The Internal Secretions in their Relations to Immunity—The Internal Secretions and the Preservation of Life.

Orthodontia and Orthopaedia of the Face.—By Victor Hugo Jackson, M. D., D. D. S., Professor of Orthodontia in the Dental Department of the University of Buffalo, etc., etc. With seven hundred and sixty original illustrations, 517 pages. Price, $5.00, net. Publishers, J. B. Lippincott Co., Philadelphia.

This work presents in detail, yet concisely and systematically the methods of the author in correcting irregularities of the teeth, together with a complete and original system for the orthopedic treatment of the face.

The author states that the principles are those applied in his own practice for many years, the results of which warrant the detailed description.

An examination of the book plainly shows the vast amount of time that Dr. Jackson has extended in research and experimentation. Probably every dentist occasionally has cases of orthodontia under treatment and this book will be found a valuable assistant.

Sunshine Thoughts for Gloomy Hours.—Prose and Verse. By George H. Chance, M. D., D. D. S. J. K. Gill Co., Portland, Ore., Publishers.

The close application and continuous confinement incident to the practice of dentistry is usually so trying to the nerves and bodily strength that few practitioners are disposed to literary work even upon technical subjects.

In this work, however, Dr. Chance has given us a very interesting little volume which will repay reading.

Christus Victor. A Student's Reverie. By Henry Nehemiah Dodge, D. D. S. Fourth edition, revised and enlarged. G. P. Putnam's Sons, New York and London, 1903.

Like the book reviewed above this is a work in the field of general literature by a member of the dental profession.

This is a remarkably well written little volume and we feel sure that it will be interesting to anybody into whose hands it falls.

Members of the profession should secure a copy—it is well gotten up and will make a most acceptable present at the Christmas period to wife, mother or sweetheart.

